# Changes in motor behavior, neuropathology, and gut microbiota of a Batten disease mouse model following administration of acidified drinking water

**DOI:** 10.1038/s41598-019-51488-z

**Published:** 2019-10-18

**Authors:** Tyler B. Johnson, Logan M. Langin, Jing Zhao, Jill M. Weimer, David A. Pearce, Attila D. Kovács

**Affiliations:** 1 Pediatrics and Rare Diseases Group, Sanford Research, Sioux Falls, South Dakota 57104 USA; 2 Population Health Group, Sanford Research, Sioux Falls, South Dakota 57104 USA; 3 Department of Internal Medicine, Sanford School of Medicine, University of South Dakota, Sioux Falls, South Dakota 57104 USA; 4 Department of Pediatrics, Sanford School of Medicine, University of South Dakota, Sioux Falls, South Dakota 57104 USA; 5 Present Address: Center for Biostatistics, Ohio State University, Columbus, Ohio 43210 USA

**Keywords:** Microbiome, Diseases of the nervous system

## Abstract

*CLN3* mutations cause the fatal neurodegenerative disorder, CLN3 Batten disease. The *Cln3*^−/−^ mouse model displays characteristic features of the human disease including motor deficits. When mice received acidified drinking water (pH 2.5–2.9) instead of normal tap water (pH 8.4) for several generations, the motor skills of *Cln3*^−/−^ mice normalized to control levels, indicating a disease-modifying effect of acidified water. Here we investigated if acidified water administered from postnatal day 21 has therapeutic benefits in *Cln3*^−/−^ mice. Indeed, acidified water temporarily attenuated the motor deficits, had beneficial effects on behavioral parameters and prevented microglial activation in the brain of *Cln3*^−/−^ mice. Interestingly, in control mice, acidified drinking water caused brain region-specific glial activation and significant changes in motor performance. Since the gut microbiota can influence neurological functions, we examined it in our disease model and found that the gut microbiota of *Cln3*^−/−^ mice was markedly different from control mice, and acidified water differentially changed the gut microbiota composition in these mice. These results indicate that acidified water may provide therapeutic benefit to CLN3 Batten disease patients, and that the pH of drinking water is a major environmental factor that strongly influences the results of murine behavioral and pathological studies.

## Introduction

Batten disease, also known as neuronal ceroid lipofuscinoses, is a group of recessively inherited, fatal lysosomal storage disorders characterized by the intracellular accumulation of autofluorescent lipopigment and progressive neurodegeneration^1^. Batten disease predominately affects children with an estimated incidence of 1–2 in 50,000 live births in the US^2^. The most common, juvenile onset form of Batten disease, CLN3 disease, is caused by mutations in *CLN3*^3^, which encodes a lysosomal/endosomal transmembrane protein of unknown function. Expression analyses indicate that the *CLN3*/*Cln3* gene and CLN3 protein is ubiquitously expressed in various human and mouse tissues^1^. CLN3 disease begins between 4 and 10 years of age with visual impairment and seizures. During the disease course, patients become blind, display motor coordination deficits and progressively lose their motor skills. The motor deterioration is accompanied by cognitive decline and patients die between 15 and 35 years of age^4^. The most common disease-causing mutation of the *CLN3* gene is a 1.02 kb deletion, which removes exons 7 and 8 and creates a premature stop codon. This mutation results in a substantial decrease in mRNA expression and stability due to nonsense-mediated decay, and most likely the mutant, truncated protein is not expressed at all^5^. The *Cln3*-knockout (*Cln3*^−/−^) mouse model of CLN3 disease displays several characteristic pathological features of the human disorder including accumulation of autofluorescent storage material in lysosomes^6^, the presence of circulating autoantibodies^7^, neurological deficits and neuropathological changes^8,9^. In a previous study using a battery of behavioral tests, we compared the *Cln3*^−/−^ and *Cln3*^*Δex7/8*^ mouse models on two different genetic backgrounds (129S6/SvEv and C57BL/6 J), males and females separately. The results showed that *Cln3*^−/−^ male mice on the 129S6/SvEv background were the most appropriate candidates for therapeutic studies. They exhibited motor deficits starting at 1 month of age in the vertical pole test and were the only mice showing impaired motor coordination in the rotarod test at both 3 and 6 months^9^.

Acidified drinking water with a pH between 2.5 and 3.0 is widely used in laboratory animal facilities to prevent the spread of pathogenic bacteria. For example, the Jackson Laboratory, a main provider of wild type and transgenic mouse strains for the research community, uses acidified drinking water^10^. The Jackson Laboratory has acidified the drinking water for their mice for more than 25 years to successfully control *Pseudomonas* species. Water in their mouse rooms is acidified with HCl to a pH of 2.5-3.0^11^. Many research institutions also use acidified drinking water in their animal facilities, including the National Institute of Health, Bethesda, MD^12–14^, Harvard Medical School, Boston, MA^15–18^, Stanford University, Stanford, CA^19–21^, Cornell University, Ithaca, NY^22–24^, University of California, Los Angeles, CA^25–27^, Boston Children’s Hospital, Boston, MA^28,29^, Texas A&M University, College Station, TX^30^, and Emory University, Atlanta, GA^31^. It is important to note that humans also regularly consume various acidic drinks^32^. Most flavored waters and sport drinks like Gatorade and PowerAde have a very low pH (pH 2.75–3.43)^32^. Similarly, most of the fruit juices and fruit drinks are acidic^32^. Additionally, most sodas including Coke and Pepsi have a pH below 3, and energy drinks, bottled teas and iced teas also have a low pH^32^. In the case of laboratory mice, the basic assumption is that acidified drinking water does not affect physiology. It has been shown, however, that acidified drinking water changes the microbial flora living in the gut and alters autoimmunity in nonobese diabetic (NOD) mice and in lupus-prone SNF1 mice^33–35^. The gut microbiota interacts with the immune system, and recent studies also indicate that changes in the gut microbiota can even influence brain function and complex behaviors^36,37^.

After our vivarium switched to acidified drinking water (pH 2.5–2.9), we noticed changes in the motor skills of *Cln3*^−/−^ mice. The motor deficit typically found in *Cln3*^−/−^ mice disappeared, the mutant mice performed similarly to healthy control mice in a pole climbing assay. This indicated a disease-modifying effect of acidified water, possibly through an influence on the composition of the gut microbiota. Here we investigated if acidified drinking water initiated at an early age (postnatal day 21) has therapeutic effects in *Cln3*^−/−^ mice from a colony that were kept on non-acidified water for generations. We found that acidified water temporarily attenuated the motor deficits, had beneficial effects on some behavioral parameters and prevented microglial activation in the thalamus, motor cortex and striatum of *Cln3*^−/−^ mice. Acidified water, however, caused brain region-specific glial activation in healthy control mice and significantly altered their motor performance. Our results also show that the gut microbiota of *Cln3*^−/−^ mice is markedly different from control mice under basal conditions and acidified drinking water differentially changed the microbiota composition between the two groups of mice. This study provides two powerful key findings. The first is that acidified water may have therapeutic effects in CLN3 Batten disease. The second is that the pH of drinking water can significantly impact a number of outcomes in even control mice, which may contribute to the inter-laboratory variations in many mouse studies. Moreover, keeping mice on acidified water may mask neurological and neuropathological phenotypes in transgenic studies.

## Results

### Acidified water administered to juvenile mice differentially changes the behavior of *Cln3*^−/−^ and wild type mice

**Figure 1:**
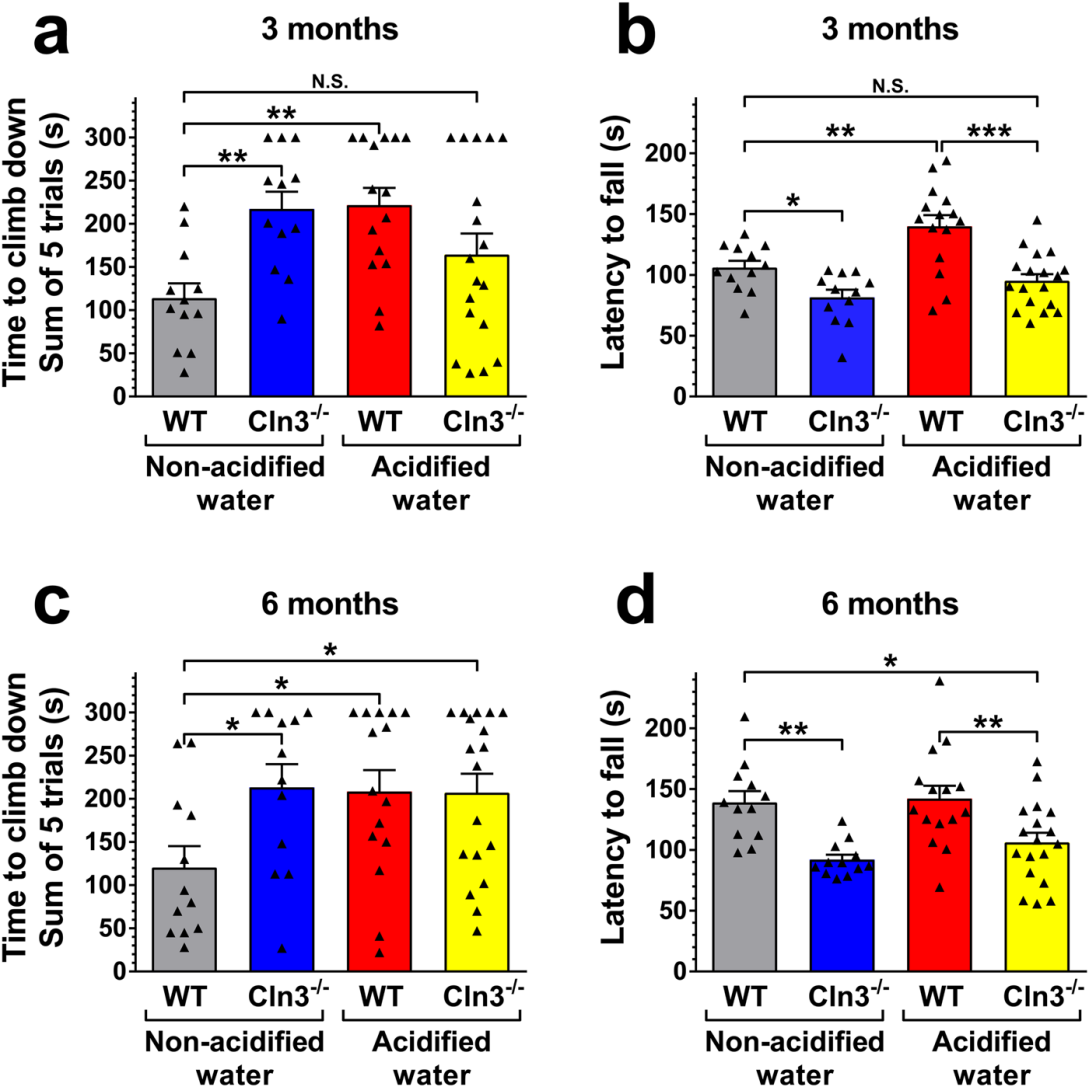
Effects of acidified drinking water on the motor function of *Cln3*^−/−^ and wild type mice. *Cln3*^−/−^ and wild type (WT) male mice either were kept on non-acidified water or received acidified water from postnatal day 21 (weaning). At the age of three and six months, mice were tested on a vertical pole to climb down (**a**,**c**), and in an accelerating rotarod test (from 0 to 48 rpm in 240 s) (**b**,**d**). Acidified water attenuated the motor deficits of *Cln3*^−/−^ mice in the pole climbing and rotarod tests at three months of age, their motor performance was not statistically different (N.S.) from that of WT mice receiving non-acidified water (**a**,**b**). The improvement was not a complete restoration to the wild type level since the difference between *Cln3*^−/−^ mice on acidified and non-acidified water did not reach statistical significance. At six month of age, however, this improvement was not be detected (**c–d**). Acidified drinking water in wild type mice impaired the pole climbing ability at three and six months (**a,c**), and enhanced the rotarod performance at three months (**b**,**d**). Columns and bars represent mean ± SEM and the symbols show the individual data (n = 12–18). Statistical significance was determined by 1-way ANOVA with Sidaks’s post-test for multiple comparisons: ^*^p < 0.05, ^**^p < 0.01, ^***^p < 0.001; N.S., not significant.

*Cln3*^−/−^ mice, similarly to patients with CLN3 disease, have motor deficits as measured in the rotarod test and a pole climbing assay^9^. After our vivarium switched to acidified drinking water and our mouse colony had received acidified drinking water for 3 generations, we tested *Cln3*^−/−^ mice and surprisingly found that their motor deficit disappeared: the pole climbing ability of *Cln3*^−/−^ mice receiving acidified drinking water was similar to previously tested wild type (129S6/SvEv) mice kept on non-acidified drinking water (Fig. S1). This indicated a disease-modifying effect of acidified water. Therefore, we tested if acidified drinking water received from an early disease stage has therapeutic effects in *Cln3*^−/−^ mice from a colony that were kept on non-acidified water for generations. *Cln3*^−/−^ and wild type mice received acidified drinking water starting at postnatal day 21 (weaning). A number of behavioral parameters were assayed at three and six months of age, the latter representing a midstage of the disease. Acidified water attenuated the motor deficits of *Cln3*^−/−^ mice at three months of age, their motor performance was statistically not different from the performance of wild type mice receiving non-acidified water in the pole climb assay and rotarod test (Fig. 1a,b). The improvement was not a complete restoration to the wild type level since the difference between *Cln3*^−/−^ mice on acidified and non-acidified water did not reach statistical significance. At six month of age, however, this improvement was not detected (Fig. 1c,d). Surprisingly, wild type mice exposed to acidified water displayed unexpected changes in pole climbing, with difficulties in climbing down the pole, frequently turning upward instead of climbing down, and scoring similar climb-down times as *Cln3*^−/−^ mice at both three and six months (Fig. 1a,c). In contrast, at the early time point in the rotarod assay, wild type mice receiving acidified water showed improved performance compared to mice on non-acidified water (Fig. 1b). This difference, however, was not maintained at the later time point (Fig. 1d).

**Figure 2:**
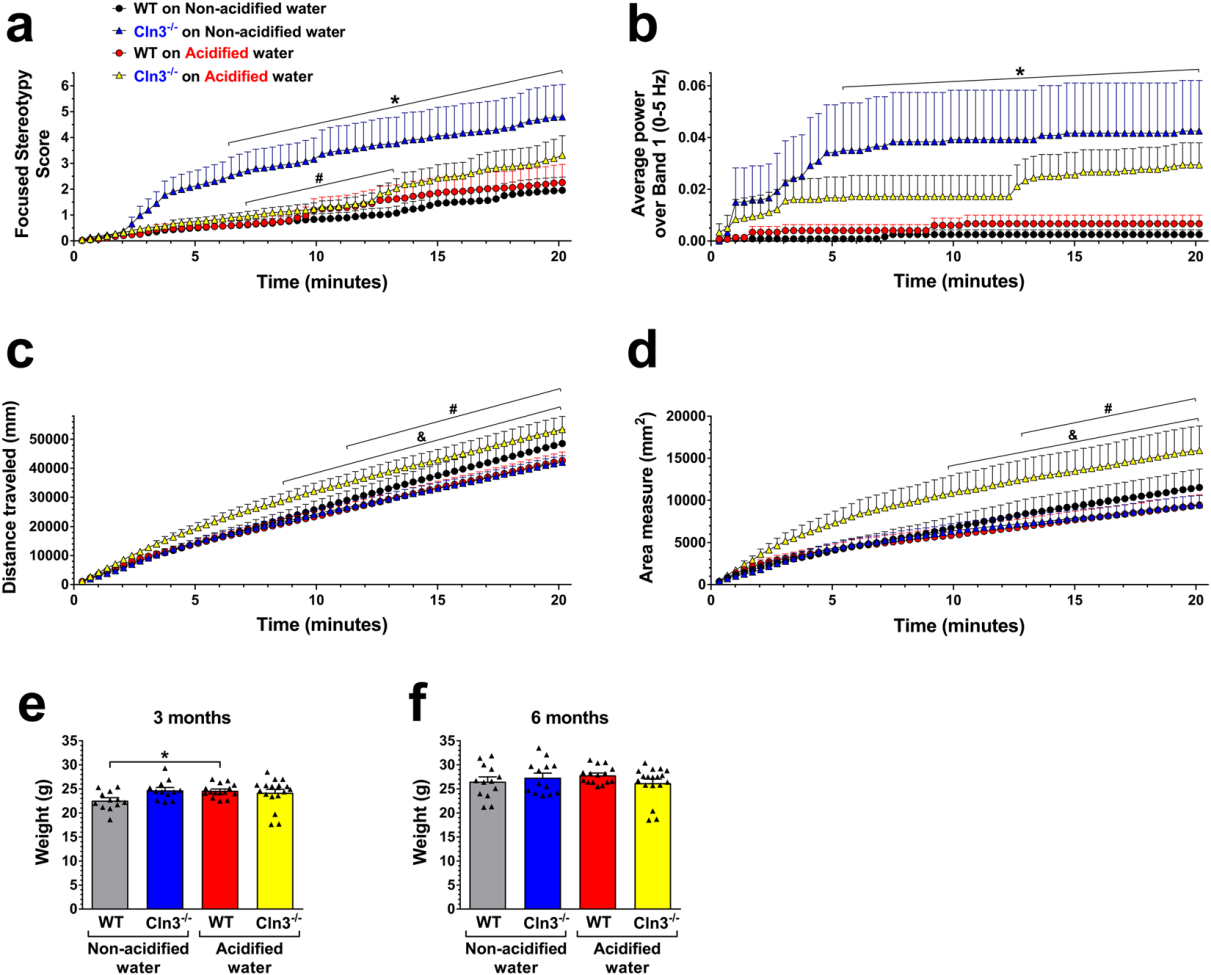
Effects of acidified drinking water on behavioral parameters of six-month-old *Cln3*^−/−^ and wild type mice measured in a force-plate actimeter. *Cln3*^−/−^ and wild type (WT) male mice either were kept on non-acidified water or received acidified water from postnatal day 21 (weaning). At six months of age, mice were tested in a force-plate actimeter, which measures several behavioral parameters in freely moving animals. The force-plate actimeter recorded data for 20.14 minutes, in fifty-nine 20.48-second frames, averaging 1,024 data points in each frame. **a,b)** In six-month-old *Cln3*^−/−^ mice on non-acidified water, the focused stereotypy score (**a**) and average power (force distribution) over band 1 (0–5 Hz) (**b**) were abnormally high and acidified water reduced them to a level that was not statistically different from that displayed by WT mice receiving non-acidified water. In the case of focused stereotypy score, the improvement reached a complete restoration to the wild type level between 7.2 and 13.3 minutes of force-plate actimeter recording when the difference between *Cln3*^−/−^ mice on acidified and non-acidified water became statistically significant. The focused stereotypy score quantifies vertical activity including head bobbing, grooming, rearing and scratching, and the average power was calculated by fast-Fourier transformation on force variation. **c**,**d)** In six-month-old *Cln3*^−/−^ mice, acidified water markedly increased the distance traveled (**c**) and the area covered (**d**). (**e**,**f**) Weights of the mice assessed in the behavioral tests at three (**e**) and six months (**f**) of age. Symbols and bars in graphs (**a**,**d**) represent mean ± SEM (n = 12–18). Columns and bars in graphs (**e,f**) represent mean ± SEM and the symbols show the individual data (n = 12–18). Statistical significance for the force-plate actimeter data was determined by repeated measures 2-way ANOVA with Dunnett’s post-test (**a-d**): ^*^p < 0.05, *Cln3*^−/−^ on Non-acidified vs. WT on Non-acidified; ^#^p < 0.05, *Cln3*^−/−^ on Non-acidified vs. *Cln3*^−/−^ on Acidified; ^&^p < 0.05, *Cln3*^−/−^ on Acidified vs. WT on Acidified. Weights of the mice were analyzed by 1-way ANOVA with Sidaks’s post-test (**e**,**f**): *p < 0.05.

To further monitor behavioral parameters in freely moving animals, a force-plate actimeter was used. The force-plate actimeter recorded data for 20.14 minutes, in fifty-nine 20.48-second frames, averaging 1,024 data points in each frame. At six months of age, the total distance traveled, area covered, spatial statistic (percentage of the platform area used for moving), bout of low mobility and number and degree of left and right turns were similar in wild type and *Cln3*^−/−^ mice kept on non-acidified water (Fig. 2 and data not shown). The focused stereotypy score and average power (force distribution) over band 1 (0–5 Hz) of *Cln3*^−/−^ mice, however, were abnormally high, and acidified water reduced these behavioral parameters to a level that was not statistically different from that displayed by wild type mice receiving non-acidified water (Fig. 2a,b). In the case of focused stereotypy score, the improvement reached a complete restoration to the wild type level between 7.2 and 13.3 minutes of force-plate actimeter recording when the difference between *Cln3*^−/−^ mice on acidified and non-acidified water became statistically significant. The force-plate actimeter also revealed that acidified drinking water selectively increased the locomotor activity of six-month-old *Cln3*^−/−^ mice: the distance traveled and the area covered were both markedly increased (Fig. 2c,d). Acidified water had no effect on the spatial statistic, bout of low mobility and number and degree of left and right turns in *Cln3*^−/−^ or wild type mice (data not shown). The force-plate actimeter did not detect any behavioral anomalies in three-month-old *Cln3*^−/−^ mice, regardless to which water they received (Fig. S2 and data not shown).

To confirm that weight was not a confounding factor in the differences observed in motor performance, we monitored body weight throughout this study. Comparing the weights of the various groups of mice the only difference was found between wild type mice at three months of age, with mice receiving acidified drinking water being heavier (Fig. 2e,f).

### Acidified water prevents microglial activation in the thalamus, motor cortex and striatum of *Cln3*^−/−^ mice

**Figure 3:**
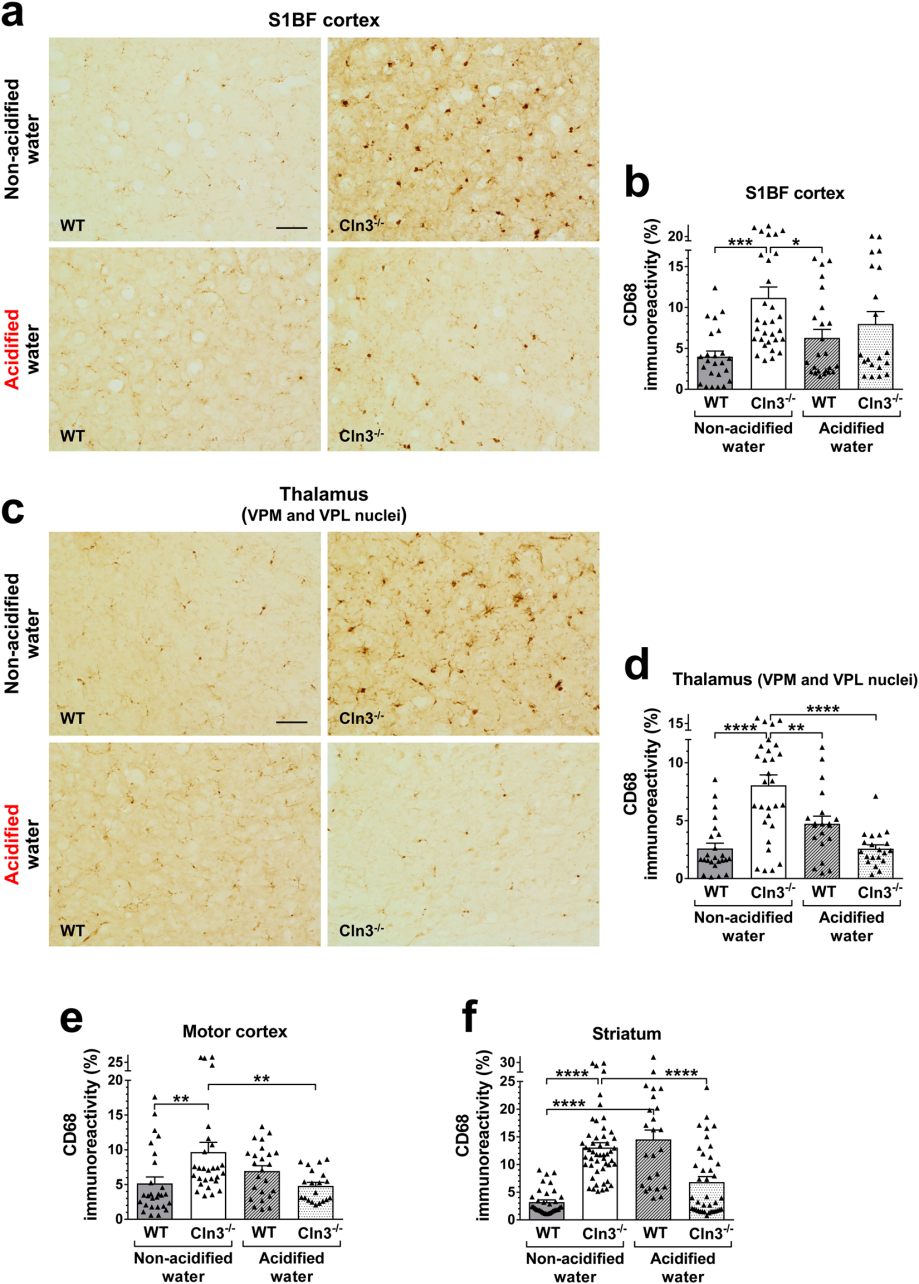
Acidified drinking water prevents microglial activation in the thalamus, motor cortex and striatum of *Cln3*^−/−^ mice. *Cln3*^−/−^ and wild type (WT) male mice either were kept on non-acidified water or received acidified water from postnatal day 21 (weaning). At six months of age, activated microglia was detected in the brain by immunohistochemical staining for CD68. Quantitative image analysis was performed in two sensory brain regions prominently affected by the disease, somatosensory barrel field (S1BF) cortex (**a**,**b**) and ventral posteromedial (VPM)/ventral posterolateral (VPL) nuclei of the thalamus (**c**,**d**), and also in the motor cortex and striatum (**e**,**f**), two regions involved in motor control. In *Cln3*^−/−^ mice, acidified water prevented microglial activation in the thalamus, motor cortex and striatum, and attenuated it in the S1BF cortex (CD68 immunoreactivity was not statistically different from that in wild type mice on non-acidified water). In WT mice, acidified water caused significant microglial activation in the striatum. Data are plotted as percent area of CD68 immunoreactivity. Columns and bars represent mean ± SEM and the symbols show the individual data (20–56 fields from 3 mice in each experimental group). Statistical significance was determined by 1-way ANOVA with Sidak’s post-test: ^*^p < 0.05, ^**^p < 0.01, ^***^p < 0.001, ^****^p < 0.0001. The scale bars in the images represent 50 µm.

**Figure 4:**
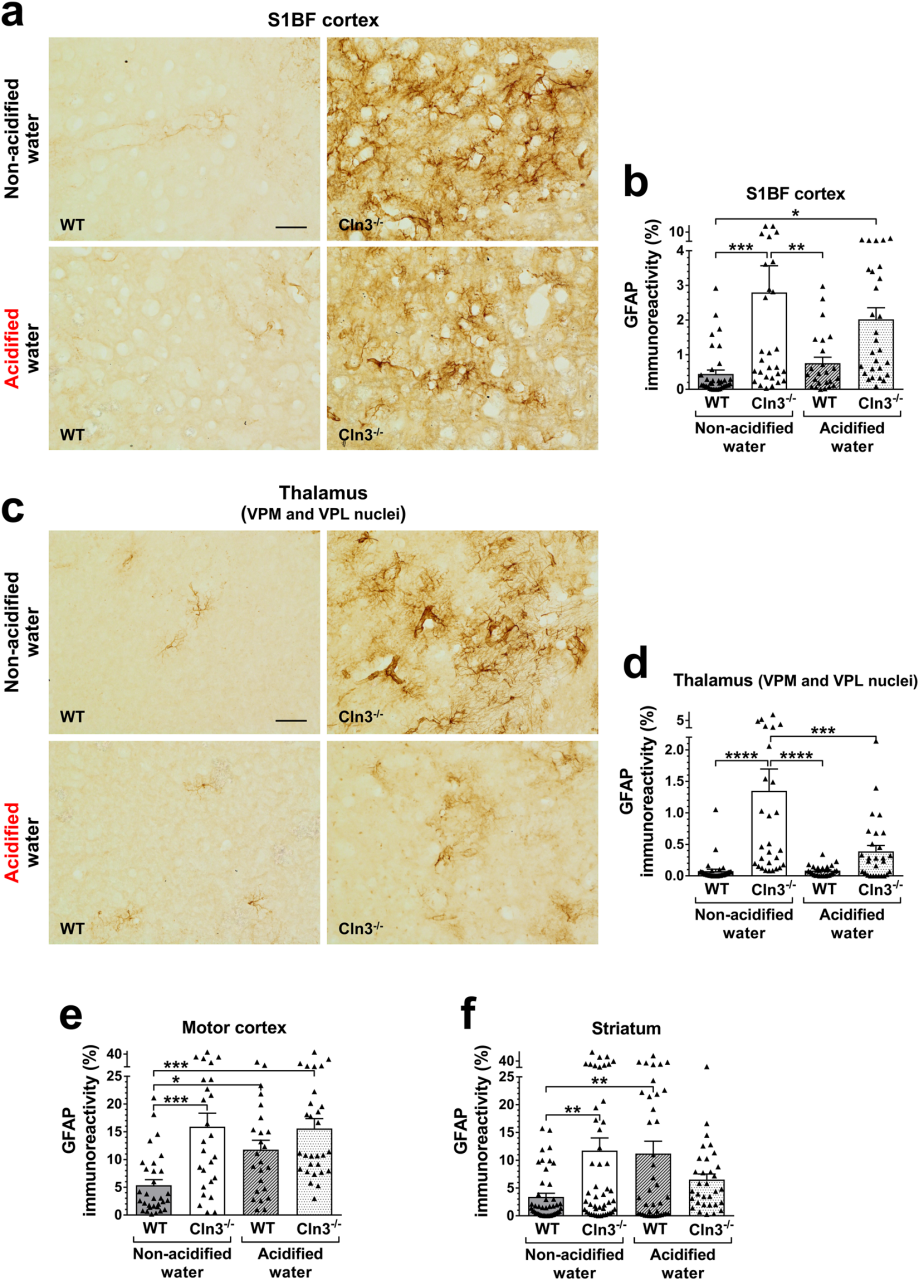
Acidified drinking water prevents astrocytosis in the thalamus of *Cln3*^−/−^ mice. *Cln3*^−/−^ and wild type (WT) male mice either were kept on non-acidified water or received acidified water from postnatal day 21 (weaning). At six months of age, astrocytosis (activation of astrocytes) was detected in the brain by immunohistochemical staining for the glial intermediate filament protein, GFAP. Quantitative image analysis was performed in two sensory brain regions prominently affected by the disease, somatosensory barrel field (S1BF) cortex (**a**,**b**) and ventral posteromedial (VPM)/ventral posterolateral (VPL) nuclei of the thalamus (**c**,**d**), and also in the motor cortex and striatum (**e**,**f**), two regions involved in motor control. In *Cln3*^−/−^ mice, acidified water prevented astrocytosis in the thalamus, and attenuated it in the striatum (GFAP immunoreactivity was not statistically different from that in wild type mice on non-acidified water). In wild type mice, acidified water caused significant astrocytosis in the motor cortex and striatum. Data are plotted as percent area of GFAP immunoreactivity. Columns and bars represent mean ± SEM and the symbols show the individual data (24–48 fields from 3 mice in each experimental group). Statistical significance was determined by 1-way ANOVA with Sidak’s post-test: ^*^p < 0.05, ^**^p < 0.01, ^***^p < 0.001, ^****^p < 0.0001. The scale bars in the images represent 50 µm.

Accumulation of lysosomal storage material and activation of astrocytes and microglia are early neuropathological hallmarks of CLN3 Batten disease that are recapitulated in *Cln3*^−/−^ mice^8,38^. We quantified these markers at six months of age in two sensory brain regions prominently affected by the disease, somatosensory barrel field (S1BF) cortex and ventral posteromedial (VPM)/ventral posterolateral (VPL) nuclei of the thalamus, and also in the motor cortex and striatum, two regions involved in motor control. Lysosomal storage material was detected immunohistochemically using an antibody to subunit c of the mitochondrial ATP synthase, a major component of the storage material in CLN3 disease^39^. Administration of acidified water had no effect on the accumulation of lysosomal storage material in the cortex, thalamus or striatum of 6-month-old *Cln3*^−/−^ mice (Fig. S3). Acidified water, however, attenuated microglial activation in the S1BF cortex and prevented it in the thalamus, motor cortex and striatum (Fig. 3). Acidified water also attenuated astrocytosis in the striatum of 6-month-old *Cln3*^−/−^ mice and prevented it in the thalamus (Fig. 4). In wild type mice, acidified water caused pronounced microglial activation in the striatum (Fig. 3f) and significant astrocytosis in the motor cortex and striatum (Fig. 4e,f). Acidified water did not trigger accumulation of lysosomal storage material in the thalamus, cortex or striatum of wild type mice (Fig. S3).

### The gut microbiota compositions of *Cln3*^−/−^ and wild type mice are markedly different and acidified water differentially changes them

**Figure 5:**
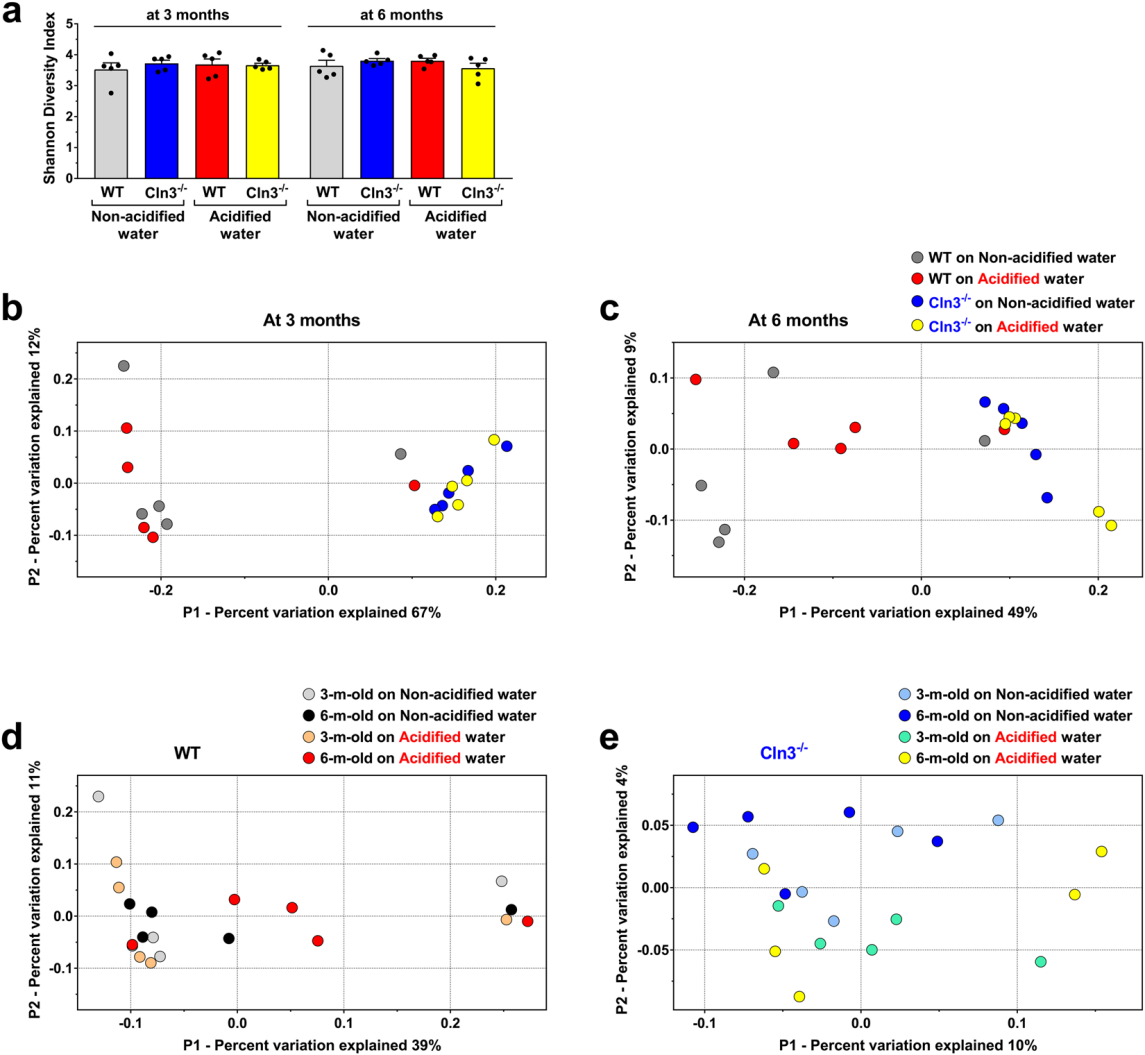
The gut microbiota of *Cln3*^−/−^ and wild type mice are markedly different. *Cln3*^−/−^ and wild type (WT) male mice either were kept on non-acidified water or received acidified water from postnatal day 21 (weaning). Fecal pellets were collected at three and six months of age to analyze the gut microbiota by 16S rRNA gene sequencing. (**a)** Alpha diversity, the microbial diversity within a sample quantified by the Shannon diversity index, was very similar in *Cln3*^−/−^ and wild type mice independently of the age or type of drinking water. Columns and bars represent mean ± SEM and the symbols show the individual data (n = 5 mice, from 4–5 different cages for each group). (**b**,**e)** Comparison of the overall gut microbiota compositions in the different groups (beta diversity) by principal coordinates analysis. Each symbol represent an individual mouse (n = 5 mice, from 4–5 different cages for each group). (**b**,**c)** The bacterial community structure in *Cln3*^−/−^ and wild type mice receiving non-acidified water was markedly different at three and six months of age (p = 0.012 and 0.008) and acidified water did not affect this difference (p = 0.005 at 3 months and p = 0.008 at 6 months). (**d**,**e)** Acidified water and aging did not result in statistically significant alterations in the overall gut microbiota composition in *Cln3*^−/−^ and wild type mice. Statistical significance in the beta diversity analyses was determined by the nonparametric statistical method “adonis” in R package “vegan”.

Since the gut microbiota can influence neurological functions, we examined the gut flora of *Cln3*^−/−^ and wild type mice on acidified and non-acidified drinking water using fecal samples. Fecal pellets were collected during the pole climbing test and the force-plate actimeter sessions. The microbiota composition of fecal samples was determined by MR DNA (www.mrdnalab.com, Shallowater, TX, USA) using 16 S rRNA gene sequencing. Alpha diversity, the microbial diversity within a sample, was quantified by the Shannon diversity index, which relates both taxonomic richness and evenness^40^. Alpha diversity was very similar in *Cln3*^−/−^ and wild type mice independently of the age or type of drinking water (Fig. 5a). To compare the overall gut microbiota compositions in the different groups (beta diversity), principal coordinates analysis was conducted using the “phyloseq” package under R software^41^. There was a statistically significant difference in the overall microbiota composition between *Cln3*^−/−^ and wild type mice on non-acidified water at three and six months of age and acidified water did not affect this difference (Fig. 5b,c). Acidified water and aging did not result in statistically significant alterations in the bacterial community structure in wild type or *Cln3*^−/−^ mice (Fig. 5d,e).

**Figure 6:**
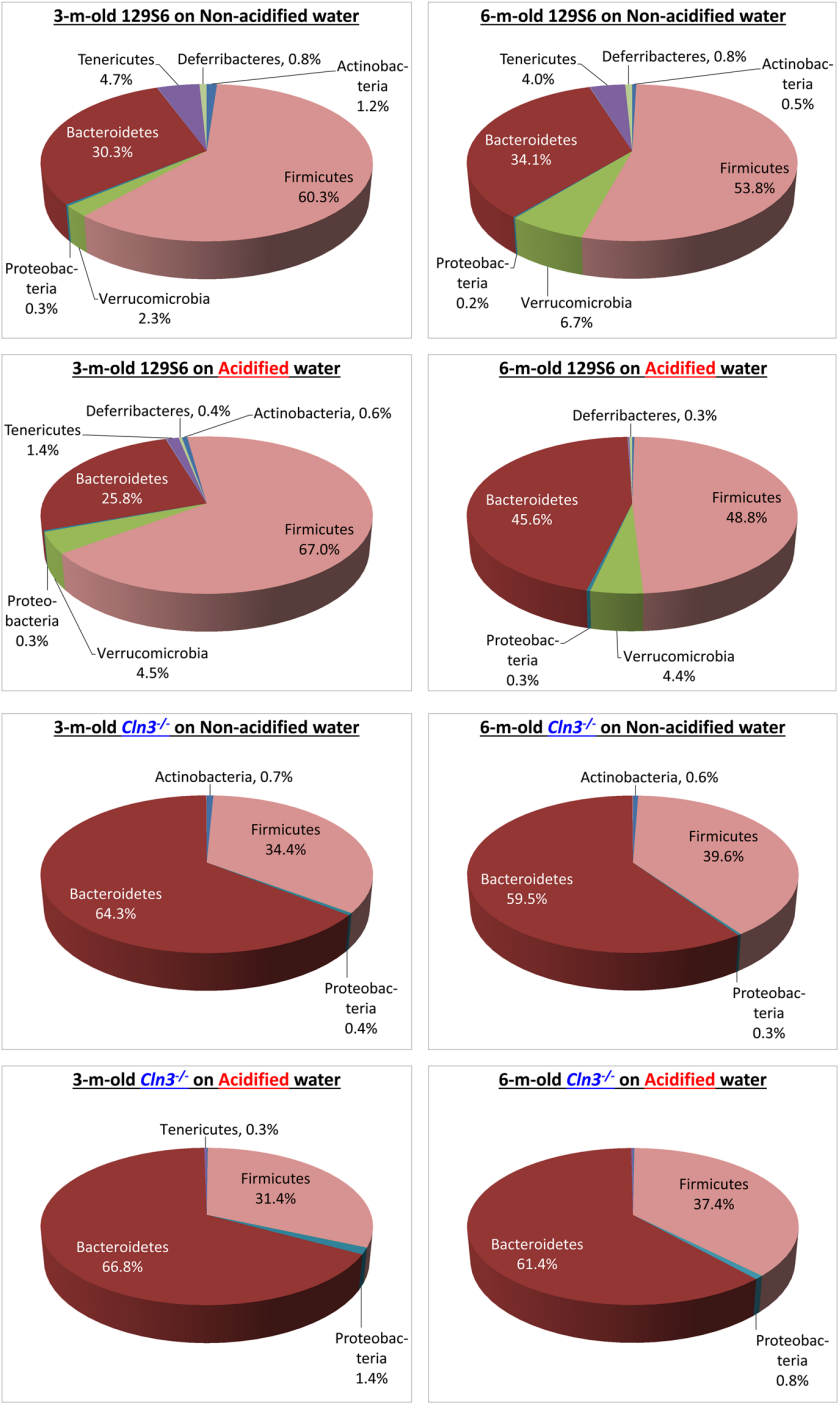
Representation of bacterial phyla in the gut microbiota of *Cln3*^−/−^ and wild type mice kept on non-acidified water or receiving acidified water from postnatal day 21. *Cln3*^−/−^ and wild type (WT) male mice either were kept on non-acidified water or received acidified water from postnatal day 21 (weaning). Fecal pellets were collected at three and six months of age to analyze the gut microbiota by16S rRNA gene sequencing. The pie charts show the percent composition of the gut microbiota at the phylum taxonomic level (averaged from 5 mice for each group).

**Figure 7:**
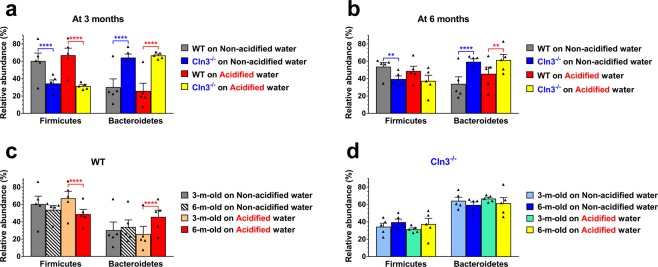
Phylum level analysis of the gut microbiota of *Cln3*^−/−^ and wild type mice kept on non-acidified water or receiving acidified water from postnatal day 21. *Cln3*^−/−^ and wild type (WT) male mice either were kept on non-acidified water or received acidified water from postnatal day 21 (weaning). Fecal pellets were collected at three and six months of age to analyze the gut microbiota by16S rRNA gene sequencing. (**a**,**b)** The gut microbiota of *Cln3*^−/−^ wild type mice at the phylum taxonomic level are dramatically different at both three (**a**) and six months (**b**) of age. (**c)** Acidified water caused age-dependent changes (from three to six months) in the phylum composition of the gut microbiota in wild type mice. (**d)** Lack of age-dependent changes (from three to six months) in the phylum composition of the gut microbiota in *Cln3*^−/−^ mice. Columns and bars represent mean ± SEM and the symbols show the individual data (n = 5 mice). Statistical significance was determined by 2-way ANOVA with Bonferroni’s post-test for multiple comparisons: ^**^p < 0.01; ^****^p < 0.0001.

Analysis of the gut microbiota composition of *Cln3*^−/−^ and wild type mice at the individual taxonomic levels revealed a number of differences. *Firmicutes* and *Bacteroidetes* are the two major phyla in the human and mouse gut microbiota. On non-acidified drinking water, while the *Firmicutes* phylum dominated the gut microbiota of wild type mice at both three and six months of age (60.3% and 53.8%), *Bacteroidetes* was the main phylum in *Cln3*^−/−^ mice at both time points (64.3% at 3 months and 59.5% at 6 months) (Figs 6 and 7a,b). Acidified water administered from postnatal day 21 did not change these differences by three months of age. At six months, however, the proportion of *Firmicutes* became similar in wild type and *Cln3*^−/−^ mice on acidified water and the difference in *Bacteroidetes* content was less pronounced (Fig. 7a,b). This was due to a significant decrease in *Firmicutes* and increase in *Bacteroidetes* content in wild type mice by six months, whereas the phylum composition in *Cln3*^−/−^ mice on acidified water did not change from three to six months (Fig. 7c,d). The phylum composition in *Cln3*^−/−^ and wild type mice on non-acidified water was unaltered at six months as compared to three months (Fig. 7c,d).

At the class, order and family taxonomic levels, significant age-dependent differences were observed in the gut microbiota between *Cln3*^−/−^ and wild type mice on both non-acidified and acidified water (Figs S4–6). In wild type mice, acidified water caused changes in one bacterial class (*Bacteroidia*) and one order (*Bacteroidales*) at six months, in one family (*Lactobacillaceae*) at three months and in three families (*Bacteroidaceae*, *Lactobacillaceae*, *Erysipelotrichaceae*) at six months (Figs S4–6). In *Cln3*^−/−^ mice, acidified water only changed the relative abundance of one family (*Erysipelotrichaceae*) at both three and six months (Fig. S6). While significant age-dependent changes, from three to six months, were observed in the gut microbiota at the class, order and family levels in wild type mice (mainly in those receiving acidified water), age-dependent changes in *Cln3*^−/−^ mice were almost completely absent (Figs S4–6).

**Figure 8:**
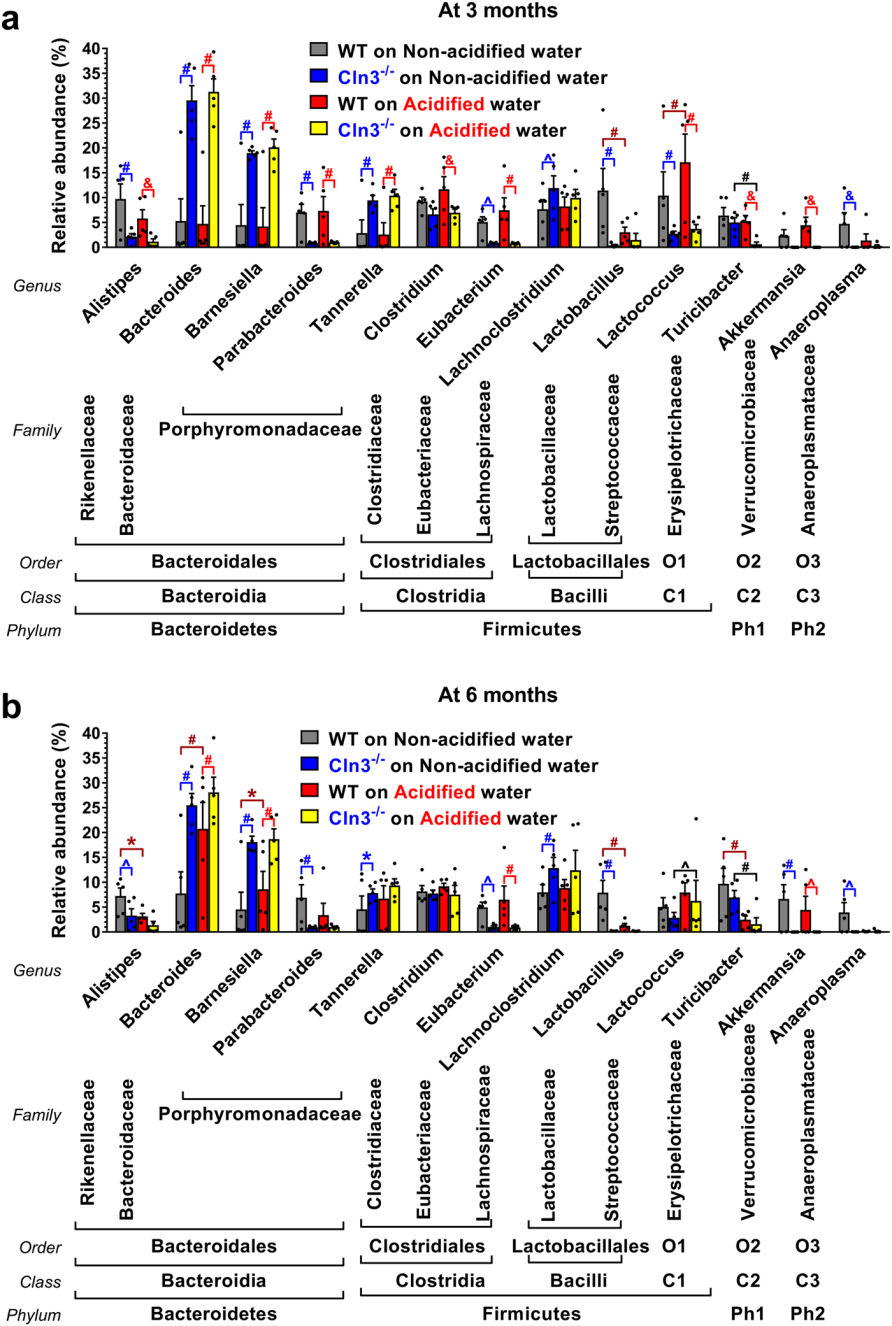
Genus level analysis of the gut microbiota in *Cln3*^−/−^ and wild type mice kept on non-acidified water or receiving acidified water from postnatal day 21. *Cln3*^−/−^ and wild type (WT) male mice either were kept on non-acidified water or received acidified water from postnatal day 21 (weaning). Fecal pellets were collected at three and six months of age to analyze the gut microbiota by 16S rRNA gene sequencing. (**a**,**b)** The gut microbiota of *Cln3*^−/−^ and wild type mice at the genus taxonomic level are markedly different at both three (**a**) and six months (**b**) of age, and acidified water differentially changes the genus composition in *Cln3*^−/−^ and wild type mice. Columns and bars represent mean ± SEM and the symbols show the individual data (n = 5 mice). Statistical significance was determined by 2-way ANOVA with Bonferroni’s post-test for multiple comparisons (^#^p < 0.0001, ^&^p < 0.001, ^^^p < 0.01, *p < 0.05; blue lines and symbols: WT vs. *Cln3*^−/−^ on Non-acidified; red lines and symbols: WT vs. *Cln3*^−/−^ on Acidified; dark red lines and symbols: WT on Non-acidified vs. WT on Acidified; black lines and symbols: *Cln3*^−/−^ on Non-acidified vs. *Cln3*^−/−^ on Acidified). Ph1, ; Ph2, *Tenericutes*; C1, *Erysipelotrichia*; C2, *Verrucomicrobiae*; C3, *Mollicutes*; O1, *Erysipelotrichales*; O2, *Verrucomicrobiales*; O3, *Anaeroplasmatales*.

At the genus level, at three months of age, pronounced differences were found between *Cln3*^−/−^ and wild type mice in the relative abundance of *Alistipes*, *Bacteroides*, *Barnesiella*, *Parabacteroides*, *Tannerella*, *Eubacterium* and *Lactococcus* on both non-acidified and acidified water, in the relative abundance of *Lachnoclostridium*, *Lactobacillus* and *Anaeroplasma* on non-acidified water only, and in the relative abundance of *Clostridium*, *Turicibacter* and *Akkermansia* on acidified water only (Fig. 8a). By six months, some of the differences observed between three-month-old *Cln3*^−/−^ and wild type mice disappeared (on non-acidified water: *Lactococcus*; on acidified water: *Alistipes*, *Parabacteroides*, *Tannerella*, *Clostridium*, *Lactococcus* and *Turicibacter*), and a new difference also emerged (on non-acidified water: *Akkermansia*) (Fig. 8b). Acidified water in wild type mice significantly altered the proportion of *Lactobacillus* and *Lactococcus* by three months of age, and of *Alistipes*, *Bacteroides*, *Barnesiella*, *Lactobacillus* and *Turicibacter* by six months of age (Fig. 8). Acidified water in *Cln3*^−/−^ mice only changed the relative abundance of *Turicibacter* at three months and of *Lactococcus* and *Turicibacter* at six months (Fig. 8). Age-dependent changes, from three to six months, in the gut microbiota composition of wild type mice at the genus level also occurred: on non-acidified water, the relative abundance of *Lactobacillus*, *Lactococcus*, *Turicibacter* and *Akkermansia* was altered; on acidified water, the relative abundance of *Bacteroides*, *Barnesiella*, *Parabacteroides*, *Tannerella* and *Lactococcus* changed significantly (Fig. S7a). In *Cln3*^−/−^ mice, there were only a few slight changes from three to six months of age: in *Bacteroides* on non-acidified water, and in *Bacteroides*, *Lachnoclostridium* and *Lactococcus* on acidified water (Fig. S7b).

Lactic acid-producing bacteria in the *Lactobacillus* genus are probiotic and provide beneficial physiological effects. In wild type mice receiving non-acidified water, the proportions of *Lactobacillus* in the gut microbiota were 11.4% and 7.9% at three and six months, whereas in *Cln3*^−/−^ mice on non-acidified water, the proportions of *Lactobacillus* were only 0.4% and 0.2% at three and six months (Fig. 8). Furthermore, acidified drinking water markedly reduced the relative abundance of *Lactobacillus* in wild type mice at both ages, to 3.0% and 1.3%, respectively (Fig. 8).

## Discussion

In *Cln3*^−/−^ mice, acidified water administered from an early postnatal age temporarily attenuated the motor deficits at three months, restored various behavioral parameters at six months and prevented microglial activation in the thalamus, motor cortex and striatum, and astrocytosis in the thalamus. Although, our study shows only limited beneficial effects of acidified drinking water on the neuropathology and neurological function in *Cln3*^−/−^ mice, any improvement is appreciated in the case of a fatal neurodegenerative disease. In CLN3 disease, synaptic dysfunction and neuronal loss progress with age. Acidified drinking water attenuated the motor deficits in *Cln3*^−/−^ mice at three months but had no effect at six months (Fig. 1), indicating that it was not able to prevent the further deterioration of neuronal pathways affecting motor function. Acidified water also provided partial protection against glial activation in the brain and reduced behavioral abnormalities as measured at six months. Altogether, these results suggest that acidified drinking water delays disease progression in *Cln3*^−/−^ mice.

In wild type mice, acidified water caused prominent alterations in the gut microbiota composition, microglial activation in the striatum and astrocytosis in the motor cortex and striatum, significantly impaired the pole climbing ability and temporarily enhanced rotarod performance. While the rotarod test measures balance and motor coordination, the pole climbing test assesses spatial orientation, balance and motor coordination. Since wild type mice on acidified water performed well in the rotarod test, their balance and motor coordination were obviously not affected. Furthermore, acidified water had no effect on the locomotor activity of wild type mice as measured in the force-plate actimeter: total distance traveled, area covered, spatial statistic (percentage of the platform area used for moving), bout of low mobility and number and degree of left and right turns were unchanged. This and the pole climbing test results suggest that acidified water impaired the spatial orientation of wild type mice, the ability to clearly differentiate between up and down. Bacteria in the *Lactobacillaceae* family and *Lactobacillus* genus have beneficial physiological and neurological effects^42^. In the gut microbiota of wild type mice receiving acidified water, there was a significant decrease in the relative abundance of the *Lactobacillaceae* family (Fig. S6) and the *Lactobacillus* genus (Fig. 8). These were the only changes that occurred at both three and six months, showing a correlation with the impairment of pole climbing ability. The acidified water-induced microglial activation in the striatum (Fig. 3f) and astrocytosis in the motor cortex and striatum (Fig. 4e,f) may also contribute to the poor pole-climbing performance of wild type mice. There was one change in the gut microbiota of wild type mice receiving acidified water that was only present at three months of age: a significant increase in the abundance of the *Lactococcus* genus (Fig. 8). This change may be linked to the superior rotarod performance of three-month-old wild type mice. *Lactococcus* species are known to produce and secrete the inhibitory neurotransmitter γ-aminobutyric acid (GABA)^43^, and it has recently been demonstrated that oral administration of GABA significantly increases the endurance of mice^44^.

Accumulated evidence indicates that the gut microbial community can undergo significant changes in neurological and neurodegenerative conditions/diseases, and a complex interaction exists between the central nervous system (CNS) and gut microbiota^37^. Our study shows that *Cln3*^−/−^ and wild type mice receiving non-acidified water have markedly different gut microbiota. At the genus level, large differences in the relative abundance of *Bacteroides*, *Barnesiella* and *Lactobacillus* were found between *Cln3*^−/−^ and wild type mice. Lactic acid-producing bacteria in the *Lactobacillus* genus are particularly important because they are probiotic, provide beneficial physiological effects and improve neurological functions in mouse disease models and in human patients^42^. The significantly reduced relative abundance of the *Lactobacillus* genus in the gut microbiota of *Cln3*^−/−^ mice on non-acidified water (Fig. 8) may contribute to the neuropathological changes and neurological deficits observed in this disease model. Changes in the gut microbiota of human patients have been reported for two neurodegenerative disorders, Parkinson’s disease^45,46^ and multiple sclerosis^47,48^. *Cln3*^−/−^ mice, similarly to multiple sclerosis patients^48^, showed a significantly decreased relative abundance of the *Lactobacillaceae* family and the *Lactobacillus* and *Parabacteroides* genera. Multiple sclerosis is an autoimmune disease and CLN3 Batten disease modelled by *Cln3*^−/−^ mice also has an autoimmune component: circulating autoantibodies against CNS proteins^7^.

It has recently been demonstrated that acidified drinking water changes the gut microbiota composition and alters autoimmunity in nonobese diabetic (NOD) mice and in lupus-prone SNF1 mice^33–35^. Acidified water can influence the gut microbiota in two ways. First, acidified water definitely changes the oral and esophageal microbiota resulting in an altered microbial input to the stomach and intestine. Second, it was recently shown that acidified water significantly lowered the pH in the duodenum, jejunum, cecum, and colon of non-obese diabetic (NOD/ShiLtJ) mice, and the lower pH was accompanied with dramatic alterations in the gut microbiota^35^. In another study, though Sofi *et al*.^33^ did not find acidified water-induced significant pH changes in the gastrointestinal tract of non-obese diabetic mice, they measured the pH without fasting the mice and the food in the gastrointestinal tract most likely diminished the pH differences. The pH in the mouse stomach is 4.0 at fasting and 3.0 after feeding^49^. Considering the small, ~0.4-ml capacity of the mouse stomach^49^ and the ~5.8-ml daily water intake of a mouse^50^, acidified water with a pH of 2.5–2.9 can definitely induce at least temporary pH changes in the stomach and further in the small and large intestines.

The relative resistance of the gut microbiota to acidified water-induced changes in *Cln3*^−/−^ mice compared to wild type mice is surprising. In the gastrointestinal tract of adult mice, *Cln3* has a detectable, low-level expression only in the colon^51^ and in colonic macrophages^52^. The slight change in the gut microbiota by acidified drinking water in *Cln3*^−/−^ mice suggests that the lack of *Cln3* expression in the gastrointestinal tract, particularly in the colon, stabilizes the gut microbiota composition. The *Cln3* gene encodes a lysosomal/endosomal transmembrane protein, CLN3, and the exact function of CLN3 is still unknown. Studies in yeast, mice, and human cells suggest a role of CLN3 in lysosomal pH regulation, autophagy, endocytosis, protein transport from the trans-Golgi, and proliferation^1^. Which of the suggested CLN3 functions are important in the interaction between intestinal bacteria and the gastrointestinal tract is currently unclear and needs further investigation. Since we determined the microbiota composition in fecal pellets which mainly represent the microbiota of the large intestine (cecum and colon), and the gut microbiota changes remarkably along the length of the gastrointestinal tract^53,54^, it is possible that acidified water induced more pronounced changes in the small intestine microbiota composition of *Cln3*^−/−^ mice. Acidified water, by decreasing the pH and increasing chloride concentration in the intestinal lumen, may affect intestinal physiology independently of the gut microbiota and this can be a contributing factor to the behavioral changes observed in *Cln3*^−/−^ and wild type mice. For example, chemosensitive vagus nerve afferents in the intestine are activated in response to luminal pH, osmolality and chemical stimulation^55^. The vagal afferent pathway is involved in the activation/regulation of the hypothalamic–pituitary–adrenal axis^56^, and thus able to influence behavior. Moreover, it has been shown that the vagus nerve can regulate the GABA neurotransmitter pathway in the cortex and hippocampus^57,58^. Acidified water may also modulate the function of intestinal enteroendocrine cells, which release over 30 gastrointestinal neurohormones. Following their release, gastrointestinal neurohormones enter the bloodstream and several of them act centrally to modulate the activity of brainstem vagal neurons^55^.

Our results show that acidified drinking water had quite different effects on motor function and glial activation in *Cln3*^−/−^ and wild type mice. The differential effects may be due to several previously discussed factors such as the markedly different gut microbiota, the significant alterations in the gut microbiota of wild type mice by acidified water, and the function of CLN3 in intestinal and glial physiology. Future metabolomic analysis and cytokine profiling of small intestine, fecal, serum and brain region samples will identify pathways and mechanisms of how acidified drinking water alters the physiology and neurological functions of *Cln3*^−/−^ and wild type mice.

In summary, our results in *Cln3*^−/−^ mice suggest that acidified drinking water may have beneficial effects for CLN3 Batten disease patients. The acidified water-induced changes in glial activation, gut microbiota composition and behavior of *Cln3*^−/−^ and wild type mice indicate that the pH of drinking water is an environmental factor that strongly influences the results of murine behavioral and pathological studies.

## Methods

### Animals

129S6/SvEv wild type and *Cln3*^−/−^ (129S6/SvEv) mice were maintained in our mouse colony and were kept on non-acidified drinking water for generations. *Cln3*^−/−^ mice were originally backcrossed with 129S6/SvEv mice for 12 generations and maintained as a homozygous *Cln3*^−/−^ colony. All mice were housed in the same room with a 14-h light, 10-h dark cycle. Mice were housed in ventilated microisolator cages (4–5 mice/cage) with ad libitum access to food and water. Mice were fed with the Teklad Global 2918 diet (Harlan Laboratories, Indianapolis, IN), and their drinking water was non-acidified tap water (pH 8.4). Groups of *Cln3*^−/−^ and wild type mice received acidified drinking water from postnatal day 21 (weaning). Acidified drinking water was prepared by acidifying tap water with HCl to pH 2.5–2.9 (average was 2.8) using a Technilab BMI BV water acidification system (Tecniplast USA, West Chester, PA). Male mice were used in the behavioral experiments and in the neuropathological and gut microbiota analyses. All animal procedures were carried out according to the guidelines of the Animal Welfare Act and NIH policies, and were approved by the Sanford Research Animal Care and Use Committee.

### Behavioral testing

All behavioral testing occurred during the light phase. Mice (all male) were transported to the behavioral testing room where the lights had been dimmed. The lights had a dimmer switch and the dimmer slider was always set to the middle position, decreasing the brightness at the vertical pole to 60 lux as measured by the Lux Meter android application by My Mobile Tools Dev. Mice were labeled on their tails with a marker for easy identification, weighed, and were allowed to acclimatize to the room at least for 20 minutes before starting the behavioral tests. All mice were tested first in the pole climbing test and then in the force-plate actimeter. One day later, the same mice were also tested in the rotarod test. The pole climbing test and rotarod test were carried out under dim light to keep the anxiety level of mice minimal. The same mice were tested at 3 and 6 months of age. To minimize the cage effect on behavior, mice from 5–6 different cages (12–18 mice) for each group were used for behavioral testing.

#### Pole climbing test

This test assesses the balance, spatial orientation, and motor coordination of mice, though anxiety may also affect the test results. The test was carried out as we previously described^9^. The vertical pole was an all-thread stainless steel rod (diameter: 1.27 cm; height: 66 cm), screwed to a 3.81-cm-thick plastic block (24.5 cm × 25.4 cm). The plastic block was covered with a 3.81-cm-thick green hunting seat cushion (nitrile rubber/PVC foam) to prevent the mice from injury when they fell from the pole. The height of the pole measured from the surface of the hunting seat cushion was 59 cm. The mouse was placed, head downward, on top of the pole, and the time until the mouse climbed down to the base of the pole was measured in 5 consecutive trials. Each climbing down trial was terminated after 60 s to avoid exhaustion. If the mouse fell from the pole, a trial result of 60 s was given. The time to climb down (sum of the 5 trials in seconds) was calculated for each mouse^9,59,60^.

#### Force-plate actimeter

The force-plate actimeter (BASi, West Lafayette, IN) measures mouse behavior with much finer spatial and temporal resolution than laser based devices. Multiple behaviors can be studied in the same freely moving animal. General locomotor activity (distance traveled, area covered, number of left and right turns), focused stereotypes (head bobbing, grooming, rearing, scratching, etc.), low mobility bouts (each defined as remaining continuously in a virtual circle of 15-mm radius for 5 s), average power (force distribution) over different frequency ranges, and tremor can be quantified^59^. Mice were placed on the 44 × 44 cm platform of the force-plate actimeter for a 20.14-min recording session, and the above-mentioned behavioral parameters were recorded. The force-plate actimeter recorded data in fifty-nine 20.48-second frames, averaging 1,024 data points in each frame. All recorded information was processed and analyzed using FPAAnalysis software version 1.10.01 (BASi, West Lafayette, IN).

#### Rotarod test

The rotarod uses a motor-driven, rotating rod to measure the fore- and hind limb motor coordination and balance of mice. Motor learning capability and endurance level may also affect the rotarod performance. The rotarod test using two Rotamex-5 accelerating rotarod instruments (Columbus Instruments, Columbus, OH, diameter of the rotating rod: 3 cm) was performed as described previously^9^. The start speed of the rotarod was 0 rpm and the acceleration was set to 0.2 rpm/s. The cut-off time was set at 240 s. Mice were trained on the rotarod in three consecutive runs. Following training, mice rested for 1.5 h and then were tested on the rotarod in three test trials each consisting of three consecutive runs, with 15 min of rest between the trials. The average latency to fall from the rotating rod in the test trials (average of the 9 runs in the 3 trials) was calculated for each mouse. Our rotarod protocol was chosen after testing different accelerations (0.1, 0.2, 0.3 and 0.4 rpm/s), end speeds (24, 36, 48, 80 rpm), and variations in the number of training and test trials^61^. One-day rotarod protocols with 0.2 rpm/s acceleration similar to ours have been used in several previous studies^62–66^. We have previously demonstrated, by measuring muscle strength, blood pH and blood levels of partial CO_2_, lactate and glucose, that our rotarod protocol does not result in fatigue^67^.

### Brain pathology

#### Histological preparation

At six months of age, one day after the rotarod test, three mice from each experimental group were perfusion-fixed with 4% paraformaldehyde. The brains were post-fixed in 4% paraformaldehyde overnight at 4 °C, cryoprotected in 30% sucrose (in PBS containing 0.05% sodium azide) at 4 °C and stored at −80 °C. For sectioning, the brains were embedded in M-1 embedding matrix and cut coronally at 35 µm on a freezing microtome using MX35 Premier Plus microtome blades (Thermo Scientific).

#### Immunohistochemistry

Three brain sections per animal were sorted, focusing on sections that contained the ventral posteromedial (VPM)/ventral posterolateral (VPL) nuclei of the thalamus, the somatosensory barrel field cortex, motor cortex and striatum, areas that are affected by the disease. Free floating brain sections were incubated in 1% hydrogen peroxide in Tris-buffered saline (TBS) for 20 min to quench endogenous peroxidase activity followed by 3 washes in TBS. Sections were then blocked in 15% goat serum-containing TBS-T (TBS with 0.3% Triton X-100) for 30 min followed by primary antibody solution overnight at 4 °C. Primary antibodies included anti-CD68 (BioRad AbD Serotec, MCA1957; 1:2000), anti-GFAP (Dako, Z0334; 1:8000), and anti-ATP synthase subunit c (Abcam, ab181243, 1:1000) diluted in TBS-T + 10% goat serum. Brain sections were washed 3 times and incubated in an appropriate secondary antibody for 2 h at room temperature. Secondary antibodies included biotinylated anti-rat and anti-rabbit IgGs (Vector Labs, BA-9400, BA-1000; 1:2000) diluted in TBS-T + 10% goat serum. Sections were washed and incubated in an ABC amplification kit (Vector Labs) for 2 h followed by 0.05% DAB solution until suitable reaction has occurred for proper signal development. Sections were then washed 3 times, mounted on microscope slides, and immersed in xylene for 10 minutes. Sections were subsequently coverslipped using DPX mounting medium and allowed to dry overnight.

#### Image acquisition analysis

All microscope slides were scanned on a Leica DM6000B slide-scanning microscope at 20X. Images were then extracted from respective regions at 2400 × 2400 pixel dimensions for image threshold analysis using ImageJ. Representative images for figures were taken on a Nikon NiE microscope.

### Analysis of the gut microbiota

#### Fecal pellet collection

Fecal pellets were collected with a pair of disinfected forceps in sterile 1.5-ml tubes at the end of the pole climbing test and the force-plate actimeter test (each mouse was tested individually). Immediately after an individual sample collection, the tube was placed on dry ice. After each mouse, the pole climbing apparatus and the platform of the force-plate actimeter were disinfected to prevent the cross-contamination of fecal samples. Collected fecal samples were stored at −80 °C, and shipped on dry ice to MR DNA (www.mrdnalab.com, Shallowater, TX, USA) for microbiota analysis.

#### 16S rRNA gene sequencing (Illumina bTEFAP 2 × 300 bp 20k Diversity assay)

DNA extraction from the mouse fecal pellets, 16S rRNA gene sequencing (Illumina bTEFAP 2 × 300 bp 20k Diversity assay) and taxonomic identification were performed by MR DNA (www.mrdnalab.com, Shallowater, TX, USA). To minimize the cage effect on the gut microbiota composition, fecal pellets collected from 4–5 different cages (5 mice) were analyzed for each group. DNA was isolated from the mouse fecal pellets using Qiagen QIAamp DNA Stool Mini Kit (Qiagen, Valencia, CA). 16S rDNA bacterial tag-encoded FLX amplicon pyrosequencing (bTEFAP), a high-throughput universal tool for bacterial diversity determination was originally described by Dowd *et al*.^68^ and has been utilized in a large number of studies analyzing the gut microbiota in different species (cattle, mice, pigs and humans) and environmental samples (see e.g.^69–71^). A redesigned modern version of bTEFAP adapted to the Illumina MiSeq platform was used to determine the microbiota composition in the mouse fecal samples.

The 16S rRNA gene V4 variable region PCR primers 515F (GTGYCAGCMGCCGCGGTAA) and 806R (GGACTACNVGGGTWTCTAAT) with barcode on the forward primer were used in a 30 cycle PCR (5 cycle used on PCR products) using the HotStarTaq Plus Master Mix Kit (Qiagen, Valencia, CA) under the following conditions: 94 °C for 3 minutes, then 28 cycles of 94 °C for 30 seconds, 53 °C for 40 seconds and 72 °C for 1 minute, followed by a final elongation step at 72 °C for 5 minutes. After amplification, PCR products were examined in a 2% agarose gel to determine the success of amplification and the relative intensity of bands. Multiple samples were pooled together (e.g., 100 samples) in equal proportions based on their molecular weight and DNA concentrations. Pooled samples were purified using calibrated Ampure XP beads. Then the pooled and purified PCR products were used to prepare DNA library according to the Illumina TruSeq DNA library preparation protocol. Sequencing was performed in an Illumina MiSeq system following the manufacturer’s guidelines. Sequence data were processed using MR DNA standardized analysis pipeline. Briefly, sequences were joined, depleted of barcodes then sequences <150 bp and sequences with ambiguous base calls were removed. Sequences were denoised, operational taxonomic units (OTUs) were generated, and chimeras were removed using the UCHIME program^72^. OTUs were defined by clustering at 3% divergence (97% similarity). Final OTUs were taxonomically classified using BLASTn against a curated database derived from GreenGenes^73^ (http://greengenes.lbl.gov/Download/), RDPII (http://rdp.cme.msu.edu) and NCBI (www.ncbi.nlm.nih.gov).

#### Data analysis

The global microbiota analyses were performed using packages under R software to identify significant differences between treatment groups based on the bacterial relative abundance profiles (beta diversity). Principal coordinates analysis was conducted using R package “phyloseq”^41^. The significance of grouping of samples was tested by nonparametric statistical method “adonis” in R package “vegan” (https://cran.r-project.org/web/packages/vegan/index.html). Post-hoc pairwise comparisons among levels of grouping factors were performed using function “pairwise.adonis” in “vegan” package with p values adjusted for multiple comparisons.

Shannon diversity index (alpha diversity) was calculated using function “estimate_richness” in the “phyloseq” R package to demonstrate the microbial diversity within the samples. Shannon indices were compared by 1-way ANOVA with Sidak’s post-test. The gut microbiota compositions at the phylum, class, order, family and genus taxonomic levels were compared by 2-way ANOVA with Bonferroni’s post-test for multiple comparisons using GraphPad Prism 7.04.

### Statistical analysis of behavioral and immunohistological data

Statistical analysis was performed using GraphPad Prism 7.04 (GraphPad Software, San Diego, CA). Data from the pole climbing test, rotarod test and weight measurement were analyzed by 1-way ANOVA with Sidak’s post-test for multiple comparisons. Data sets from the force-plate actimeter were analyzed using repeated measures 2-way ANOVA with Dunnett’s post-test for multiple comparisons. Immunohistological data were analyzed by 1-way ANOVA with Sidak’s post-test. Alpha level was 0.05 in all statistical tests.

## Supplementary information

Supplementary Figures

## Data Availability

The datasets used and/or analyzed during the current study are available from the corresponding author on reasonable request.

## Supplementary information

is avilable for this paper at 10.1038/s41598-019-51488-z.
