# Supplementary material for: Changes in motor behavior, neuropathology, and gut microbiota of a Batten disease mouse model following administration of acidified drinking water
Source: Sci Rep. 2019 Oct 18;9:14962. doi: 10.1038/s41598-019-51488-z (PMC6802212; doi:10.1038/s41598-019-51488-z)
Supplement: Supplementary file 1 — Supplementary Figures [file 41598_2019_51488_MOESM1_ESM.docx]

**Changes in motor behavior, neuropathology, and gut microbiota of a Batten disease mouse model following administration of acidified drinking water**

Tyler B. Johnson^1^, Logan M. Langin^1^, Jing Zhao^2,3^, Jill M. Weimer^1,4^, David A. Pearce^1,4^ and Attila D. Kovács^1,4,^*

^1^Pediatrics and Rare Diseases Group, ^2^Population Health Group, Sanford Research, Sioux Falls, South Dakota, 57104, USA; ^3^Department of Internal Medicine, ^4^Department of Pediatrics, Sanford School of Medicine, University of South Dakota, Sioux Falls, South Dakota, 57104, USA

***Corresponding author:**

Attila D. Kovács, PhD

Pediatric and Rare Diseases Group, Sanford Research

2301 E. 60^th^ Street N.,

Sioux Falls, South Dakota, 57104

Tel: 1-(605)312-6404

E-mail: [Attila.Kovacs@sanfordhealth.org](mailto:Attila.Kovacs@sanfordhealth.org)

**Supplementary Figures**

**Supplementary Fig. 1. *Cln3^-/-^* mice after receiving acidified drinking water for 3 generations did not display a motor deficit in the pole climbing test.** The ability of mice to climb down a vertical pole was examined according to our method. Six-month-old wild type (WT) and *Cln3^-/-^* male mice kept on non-acidified drinking water for many generations and 6-month-old *Cln3^-/-^* male mice kept on acidified drinking water for 3 generations were tested. Mice were placed, head downward, on top of the pole, and the time until they climbed down to the base of the pole was measured in 5 consecutive trials. Each climbing-down trial was terminated after 60 seconds to avoid exhaustion. The time to climb down (sum of the 5 trials in seconds) was calculated for each mouse. Columns and bars represent mean ± SEM and the symbols show the individual data (n=9-11). Statistical significance was determined by 1-way ANOVA with Sidaks’s post-test for multiple comparisons: ^*^p<0.05, ^**^p<0.01.

**Supplementary Fig. 2. The force-plate actimeter did not detect** **behavioral anomalies in three-month-old *Cln3^-/-^* mice.** *Cln3^-/-^* and wild type (WT) male mice either were kept on non-acidified water or received acidified water from postnatal day 21 (weaning). At three months of age, mice were tested in a force-plate actimeter, which measures several behavioral parameters in freely moving animals. The force-plate actimeter recorded data for 20.14 minutes, in fifty-nine 20.48-second frames, averaging 1,024 data points in each frame. **a)** Focused stereotypy score. **b)** Average power (force distribution) over band 1 (0-5 Hz). **c)** Total distance traveled. **d)** Area covered. . Symbols and bars represent mean ± SEM (n=12-18). No statistically significant differences were identified by repeated measures 2-way ANOVA with Dunnett’s post-test.

**Supplementary Fig. 3. Acidified drinking water does not affect the accumulation of lysosomal storage material in the cortex, thalamus and striatum of *Cln3^-/-^* mice.** *Cln3^-/-^* and wild type (WT) male mice either were kept on non-acidified water or received acidified water from postnatal day 21 (weaning). At six months of age, lysosomal storage material was detected in the brain by immunohistochemical staining for subunit c of the mitochondrial ATP synthase, a major component of the storage material in CLN3 Batten disease. Quantitative image analysis was performed in two sensory brain regions prominently affected by the disease, somatosensory barrel field (S1BF) cortex (**a-b**) and ventral posteromedial (VPM)/ventral posterolateral (VPL) nuclei of the thalamus (**c-d**), and also in the motor cortex and striatum (**e-f**), two regions involved in motor control. Data are plotted as percent area of subunit c immunoreactivity. Columns and bars represent mean ± SEM and the symbols show the individual data (24-56 fields from 3 mice in each experimental group). Statistical significance was determined by 1-way ANOVA with Sidak’s post-test: ^****^p<0.0001. The scale bars in the images represent 50 µm.


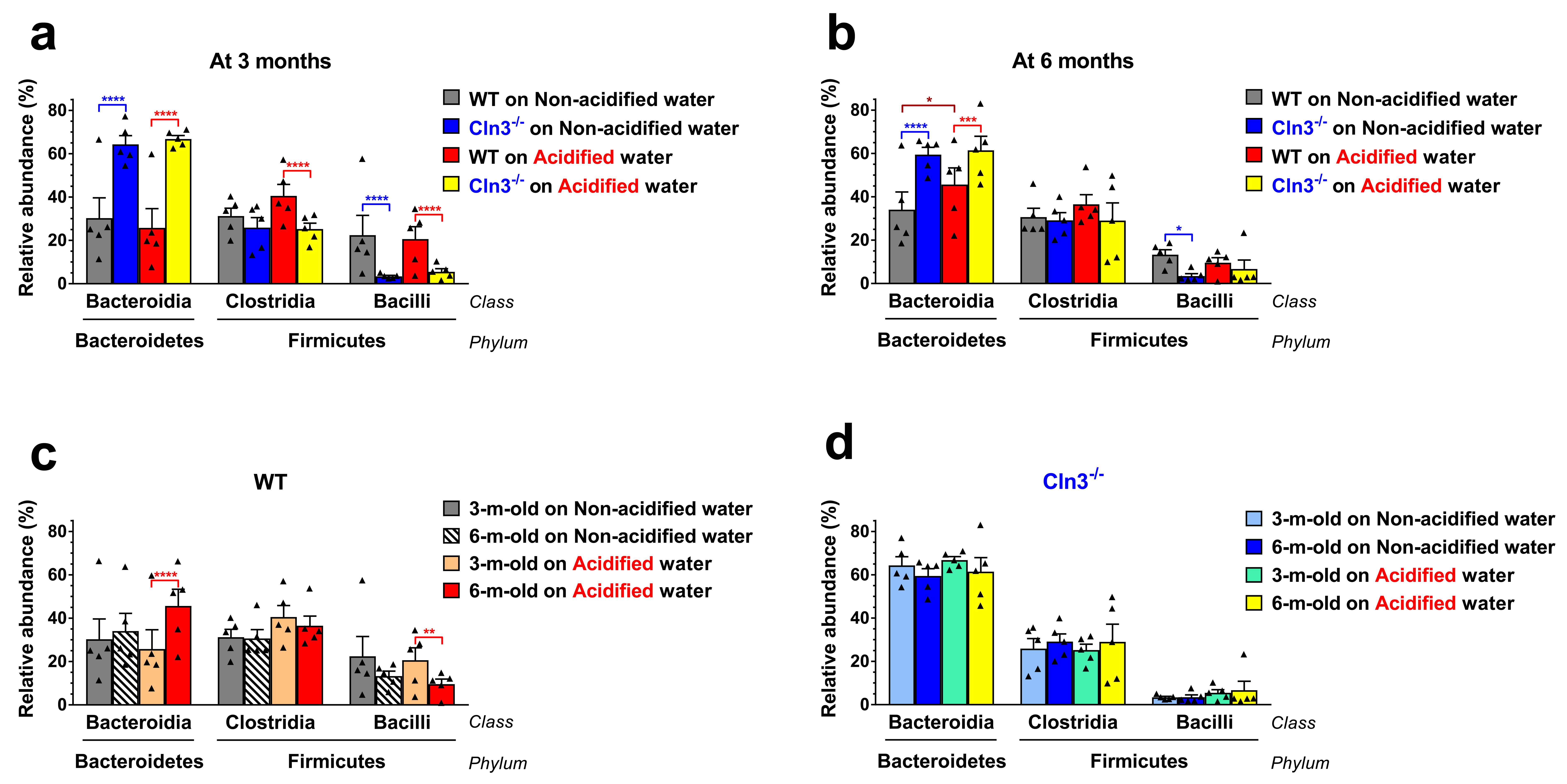


**Supplementary Fig. 4. Class level analysis of the gut microbiota of *Cln3^-/-^* and wild type mice kept on non-acidified drinking water or receiving acidified drinking water from postnatal day 21.** *Cln3^-/-^* and wild type (WT) male mice either were kept on non-acidified water or received acidified water from postnatal day 21 (weaning). Fecal pellets were collected at three and six months of age to analyze the gut microbiota by 16S rRNA gene sequencing. **a-b)** The gut microbiota of *Cln3^-/-^* and wild type mice at the class taxonomic level are markedly different at both three (a) and six months (b) of age. **c)** Acidified water caused age-dependent changes (from three to six months) in the class composition of the gut microbiota in wild type mice. **d)** Lack of age-dependent changes (from three to six months) in the class composition of the gut microbiota in *Cln3^-/-^* mice. Columns and bars represent mean ± SEM and the symbols show the individual data (n=5 mice). Statistical significance was determined by 2-way ANOVA with Bonferroni’s post-test for multiple comparisons: ^*^p<0.05, ^**^p<0.01, ^***^p<0.001, ^****^p<0.0001.

**Supplementary Fig. 5. Order level analysis of the gut microbiota of *Cln3^-/-^* and wild type mice kept on non-acidified water or receiving acidified drinking water from postnatal day 21.** *Cln3^-/-^* and wild type (WT) male mice either were kept on non-acidified water or received acidified water from postnatal day 21 (weaning). Fecal pellets were collected at three and six months of age to analyze the gut microbiota by16S rRNA gene sequencing. **a-b)** The gut microbiota of *Cln3^-/-^* and wild type mice at the order taxonomic level are significantly different at both three (a) and six months (b) of age. **c)** Acidified water caused age-dependent changes (from three to six months) in the order composition of the gut microbiota in wild type mice. **d)** Lack of age-dependent changes (from three to six months) in the order composition of the gut microbiota in *Cln3^-/-^* mice. Columns and bars represent mean ± SEM and the symbols show the individual data (n=5 mice). Statistical significance was determined by 2-way ANOVA with Bonferroni’s post-test for multiple comparisons (^**^p<0.01, ^***^p<0.001, ^****^p<0.0001). Ph1, *Bacteroidetes*; Ph2, *Tenericutes*; Ph3, *Verrucomicrobia*; C1, *Bacteroidia*; C2, *Bacilli*; C3, *Erysipelotrichia*; C4, *Clostridia*; C5, *Mollicutes*; C6, *Verrucomicrobiae*.

**Supplementary Fig. 6. Family level analysis of the gut microbiota of *Cln3^-/-^* and wild type mice kept on non-acidified water or receiving acidified water from postnatal day 21.** *Cln3^-/-^* and wild type (WT) male mice either were kept on non-acidified water or received acidified water from postnatal day 21 (weaning). Fecal pellets were collected at three and six months of age to analyze the gut microbiota by 16S rRNA gene sequencing. **a-b)** The gut microbiota of *Cln3^-/-^* and wild type mice at the family taxonomic level are markedly different at both three (a) and six months (b) of age. **c)** Acidified water caused age-dependent changes (from three to six months) in the family composition of the gut microbiota in wild type mice. **d)** Only a slight age-dependent change (from three to six months) in the family composition of the gut microbiota in *Cln3^-/-^* mice. Columns and bars represent mean ± SEM and the symbols show the individual data (n=5 mice). Statistical significance was determined by 2-way ANOVA with Bonferroni’s post-test for multiple comparisons (^#^p<0.0001, ^&^p<0.001, **^**p<0.01, *p<0.05). Ph1, *Verrucomicrobia*; C1, *Clostridia*; C2, *Erysipelotrichia*; C3, *Verrucomicrobiae*; O1, *Clostridiales*; O2, *Erysipelotrichales*; O3, *Verrucomicrobiales*.

**Supplementary Fig. 7. Age-dependent changes in the genus composition of the gut microbiota in wild type and *Cln3^-/-^* mice kept on non-acidified water or receiving acidified water from postnatal day 21.** *Cln3^-/-^* and wild type (WT) male mice either were kept on non-acidified water or received acidified water from postnatal day 21 (weaning). Fecal pellets were collected at three and six months of age to analyze the gut microbiota by 16S rRNA gene sequencing. Significant age-dependent changes (from three to six months) were found in the genus composition of the gut microbiota in wild type (**a**) and *Cln3^-/-^* mice (**b**). Columns and bars represent mean ± SEM and the symbols show the individual data (n=5 mice). Statistical significance was determined by 2-way ANOVA with Bonferroni’s post-test for multiple comparisons (^#^p<0.0001, ^&^p<0.001, **^**p<0.01, *p<0.05). Ph1, *Verrucomicrobia*; Ph2, *Tenericutes*; C1, *Erysipelotrichia*; C2, *Verrucomicrobiae*; C3, *Mollicutes*; O1, *Erysipelotrichales*; O2, *Verrucomicrobiales*; O3, *Anaeroplasmatales*
